# Trends in pulmonary tuberculosis mortality between 1985 and 2018: an observational analysis

**DOI:** 10.1186/s12890-023-02458-9

**Published:** 2023-05-26

**Authors:** Harpreet Singh, Arashdeep Rupal, Omar Al Omari, Chinmay Jani, Alaaeldin Ahmed, Shoheera Khaliqdina, Alexander Walker, Joseph Shalhoub, Carey Thomson, Dominic C. Marshall, Justin D. Salciccioli

**Affiliations:** 1 Division of Pulmonary and Critical Care Medicine, Medical College of Wisconsin, Milwaukee, WI USA; 2 Medical Data Research Collaborative, London, UK; 3 Division of Pulmonary, Critical Care Medicine, University of South Florida, Tampa, FL USA; 4 Department of Medicine, Mount Auburn Hospital, Beth Israel Lahey Health, Cambridge, MA USA; 5 Harvard Medical School, Boston, MA USA; 6 Division of Pulmonary, Critical Care & Sleep Medicine, Yale School of Medicine, New Haven, CT USA; 7 Department of Surgery and Cancer, Imperial College of London, London, UK; 8 Imperial Vascular Unit, Imperial College Healthcare NHS Trust, London, UK; 9 Division of Pulmonary and Critical Care Medicine, Mount Auburn Hospital, Beth Israel Lahey Health, Cambridge, MA USA; 10 National Heart and Lung Institute, Imperial College London, London, UK; 11 Division of Pulmonary and Critical Care, Brigham and Women’s Hospital, Boston, MA USA

**Keywords:** Tuberculosis, Mortality, WHO

## Abstract

**Background:**

Pulmonary tuberculosis (TB) is a major source of global morbidity and mortality. Latent infection has enabled it to spread to a quarter of the world's population. The late 1980s and early 1990s saw an increase in the number of TB cases related to the HIV epidemic, and the spread of multidrug-resistant TB. Few studies have reported pulmonary TB mortality trends. Our study reports and compares trends in pulmonary TB mortality.

**Methods:**

We utilized the World Health Organization (WHO) mortality database from 1985 through 2018 to analyze TB mortality using the International Classification of Diseases-10 codes. Based on the availability and quality of data, we investigated 33 countries including two countries from the Americas; 28 countries from Europe; and 3 countries from the Western Pacific region. Mortality rates were dichotomized by sex. We computed age-standardized death rates per 100,000 population using the world standard population. Time trends were investigated using joinpoint regression analysis.

**Results:**

We observed a uniform decrease in mortality in all countries across the study period except the Republic of Moldova, which showed an increase in female mortality (+ 0.12 per 100,000 population). Among all countries, Lithuania had the greatest reduction in male mortality (-12) between 1993–2018, and Hungary had the greatest reduction in female mortality (-1.57) between 1985–2017. For males, Slovenia had the most rapid recent declining trend with an estimated annual percentage change (EAPC) of -47% (2003–2016), whereas Croatia showed the fastest increase (EAPC, + 25.0% [2015–2017]). For females, New Zealand had the most rapid declining trend (EAPC, -47.2% [1985–2015]), whereas Croatia showed a rapid increase (EAPC, + 24.9% [2014–2017]).

**Conclusions:**

Pulmonary TB mortality is disproportionately higher among Central and Eastern European countries. This communicable disease cannot be eliminated from any one region without a global approach. Priority action areas include ensuring early diagnosis and successful treatment to the most vulnerable groups such as people of foreign origin from countries with a high burden of TB and incarcerated population. Incomplete reporting of TB-related epidemiological data to WHO excluded high-burden countries and limited our study to 33 countries only. Improvement in reporting is crucial to accurately identify changes in epidemiology, the effect of new treatments, and management approaches.

**Supplementary Information:**

The online version contains supplementary material available at 10.1186/s12890-023-02458-9.

## Background

Tuberculosis (TB) remains a significant cause of preventable mortality. It is the leading cause of death from a single infectious agent since 2007, ranking above human immunodeficiency virus /acquired immunodeficiency syndrome (HIV/AIDS) and the 10th leading cause of death worldwide. It is estimated that 1.4 million deaths from TB, including 0.21 million among patients with HIV infection, occurred in 2019 [1]. This death toll equals 2% of global mortality, even though it is a disease for which a cure has existed for 70 years. People in all age groups are affected by TB, but the highest burden is among adult men, who accounted for 56% of all cases in 2019, compared with 32% of cases in adult women and 12% in children [1].

The ability of the organism to efficiently establish asymptomatic, latent infection has enabled it to spread to nearly 2 billion people (about one-fourth of the world’s population) [2]. Although latent TB infection (LTBI) itself isn’t contagious, approximately 10% of persons with normal immune systems infected with *Mycobacterium tuberculosis* will develop active disease at some point in their lives without treatment. This percentage is even higher in the immunocompromised [3, 4]. During the late 1980s and the early 1990s, the number of reported TB cases increased in the industrialized world. These increases were related to the HIV epidemic, migration from countries with a high TB incidence, and the spread of multidrug-resistant TB (MDR TB). In 1993, World Health Organization (WHO) declared tuberculosis a public health emergency. Within one year, it unveiled directly observed treatment, short course, or DOTS, as its solution to the problem. Although significant progress in TB control has been achieved worldwide since then, the global burden of TB remains substantial.

The primary aim of this study is to compare mortality trends in pulmonary TB in 33 countries from the following WHO regions: Americas, Western Pacific, and Europe, from 1985–2018 and evaluate the difference in mortality trends between males and females. We have previously utilized similar methods to describe trends in mortality from cardiovascular [5] and respiratory diseases [6, 7].

## Methods

### Data sources

We utilized the WHO mortality database for the WHO member nations whose data was available. We extracted mortality data for primary respiratory TB from 1985 to 2018 using the International Classification of Diseases (ICD) -10 code A15.7. The WHO evaluates the quality of the data to ensure comparability and reliability, without adjustment for underreporting. For inclusion criteria, we first evaluated the database to check the countries with available data. Out of 194 member countries of WHO, we included countries with > 90% completeness data. We further excluded countries that either did not have five years of data or had significant breaks in data, defined as greater than three consecutive years.

Total 33 countries from WHO regions Americas, Western Pacific, and Europe met the study definition of data completeness. Among these, two countries had data available till 2018, sixteen countries had the data available till 2017, five till 2016, five till 2015, two till 2014, one country till 2013, one till 2007, and one till 2005. Region-wise, Americas included Canada and the United States (US); Europe included Austria, Belgium, Bulgaria, Croatia, Czech Republic, Denmark, Estonia, Finland, France, Germany, Greece, Hungary, Ireland, Israel, Italy, Latvia, Lithuania, Netherlands, Poland, Portugal, Republic of Moldova, Romania, Slovakia, Slovenia, Spain, Sweden, Switzerland, and United Kingdom; Western Pacific region included Australia, New Zealand, and Japan.

Crude mortality rates were dichotomized by sex and reported by year. We computed age standardized death rates (ASDRs) per 100,000 population using the world standard population. The ASDR was calculated, defined as mortality weighted to the distribution of mortality per 5-year age group, according to the WHO standard populations and world average age structure for 1998 [8]. This removes the effects of historical events on age structure and controls for differences in age structure in populations, producing age-specific mortality rates and more representative data. The estimated level of coverage for deaths with a recorded cause for death is calculated by actual reporting divided by the estimated mortality rate. Population and birth recording in all countries are specified in the data, as per the WHO standard for inclusion in the database [9]. IRB approval was not necessary as the data collected was available in the data-repositories mentioned above in a de-identified format.

### Statistical analyses

We used Joinpoint regression analysis with annualized data (between 1985 and 2018, where available) to assess changes in linear slope for mortality trends over time, as described previously [5]. In brief, Joinpoint analysis estimates the overall trends in mortality, initially with no Joinpoints, and tests for significant changes in the model with the sequential addition of Joinpoints where there is a significant change in the slope of the line. Joinpoint software (Command Line Version 4.5.0.1) is provided by the US National Cancer Institute Surveillance Research Program [10]. The model also computes an estimated annual percentage change (EAPC) for each trend by fitting a regression line to the natural logarithm of the rates. Mortality data were missing in a small subset of countries in the WHO mortality database for one to five calendar years. Joinpoint software requires continuous data throughout the observation period to be suitable for analysis. Therefore, for the purpose of Joinpoint analysis only, we imputed using a last observation carried forward for countries with missing data. If a country had more than three consecutive years of missing data during the observation period, it was excluded from the analysis to avoid excess imputation. There were no other modifications to the data. Changes in ASDR over the observation period are calculated as crude absolute differences between first and last data points for the earliest and most recent years available.

## Post-hoc analysis

Acknowledging reviewers remarks and re-evaluating our primary analysis, we reported Gross Domestic Product (GDP) per capita (current US dollars), heath expenditure per capita (current US dollars), and Socio-demographic Index (SDI) for each country. We utilized Word Bank datafiles to extract GDP and per capita heath expenditure, and Global Burden of Disease (GBD) dataset to extract SDI. Linear regression analysis was performed to calculate correlation between TB-related ASDR in each country with their respective GDP, health expenditure, and SDI. Pearson correlation coefficient along with *p*-values were reported. In addition, we calculated the mean and standard deviation for each variable for countries with ASDRs > 1 per 100,000 population as the end of the study period in males and > 0.25 in females with those with ASDR < 1 and < 0.25 in males and females respectively. To compare the two cohorts, statistical analysis of all three variables was performed using t test and p values were reported. Analysis was performed using SPSS 26.0 (SPSS Inc, Chicago, IL).

## Results

### Current mortality for TB

**Fig. 1:**
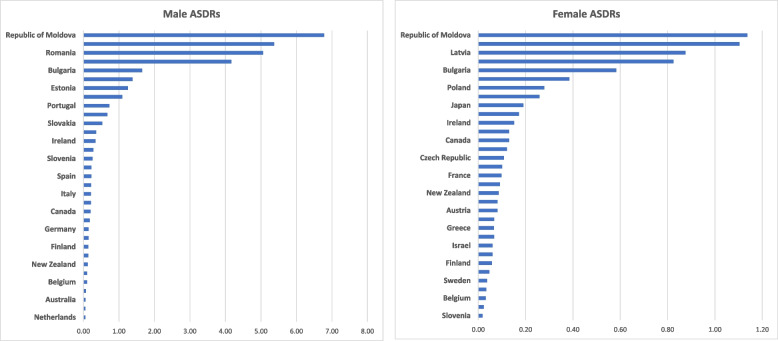
**A** & **B **Tuberculosis related age standardized death rates per 100,000 population for males and females in last year of study period

Table 1 and Fig. 1A and B show the most recent calendar year mortality data. In the Americas region, only US and Canada were met inclusion definition for data completeness. Canada had ASDR of 0.20 per 100,000 population in males and 0.13 in females in 2005, whereas the US had ASDR of 0.13 in males and 0.07 in females in 2007. In the Europe region, the Republic of Moldova had the highest ASDR in 2018 for both males (6.78) and females (1.14). Switzerland had the lowest ASDR in 2013 for males (0.05) shared with the Netherlands in 2017. Switzerland also had the lowest ASDR in 2013 for females (0.02) shared with Slovenia in 2017. In the Western Pacific region, Japan had the highest ASDR for both males (0.67) and females (0.19) in 2017, while Australia had the lowest ASDR for both males (0.06) and females (0.07) in 2017.

**Table 1:**
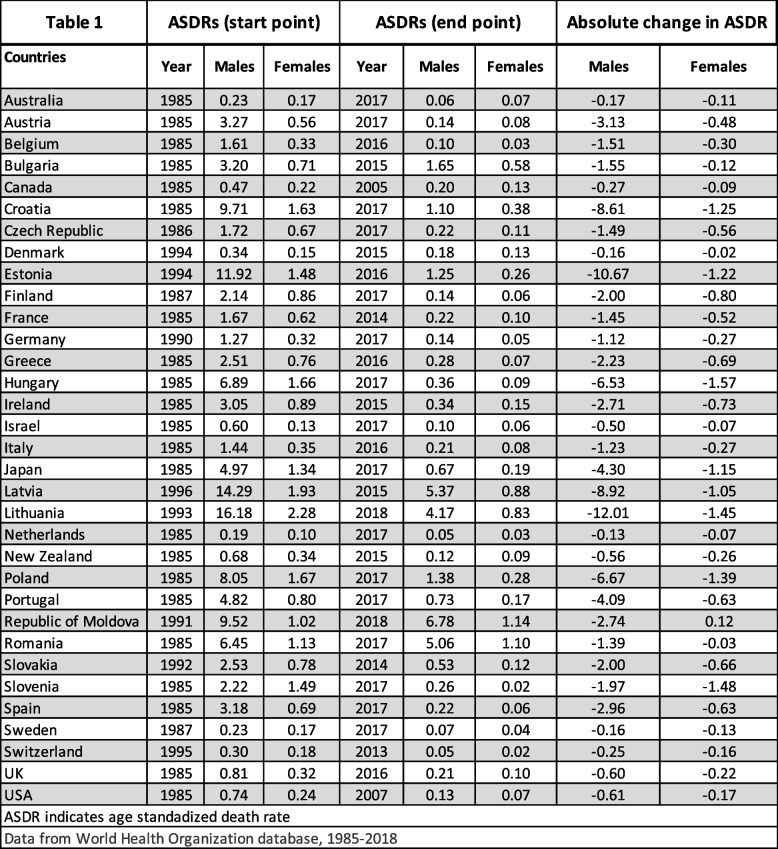
Tuberculosis related age standardized death rates per 100,000 population for males and females

### Changes in pulmonary TB-related mortality between start and end points

**Fig. 2:**
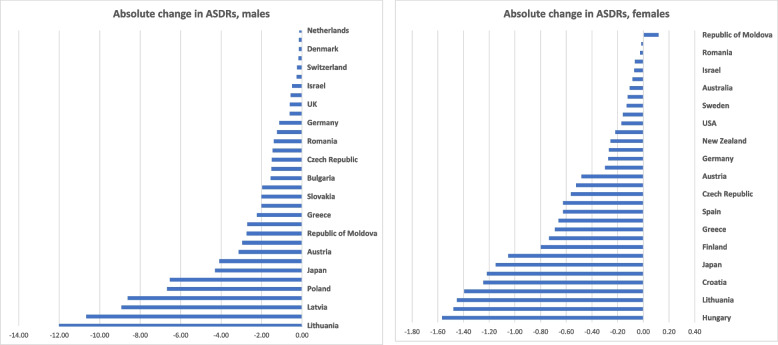
**A** & **B **Absolute change in Tuberculosis related age standardized death rates per 100,000 population in males and females over study period

**Fig. 3:**
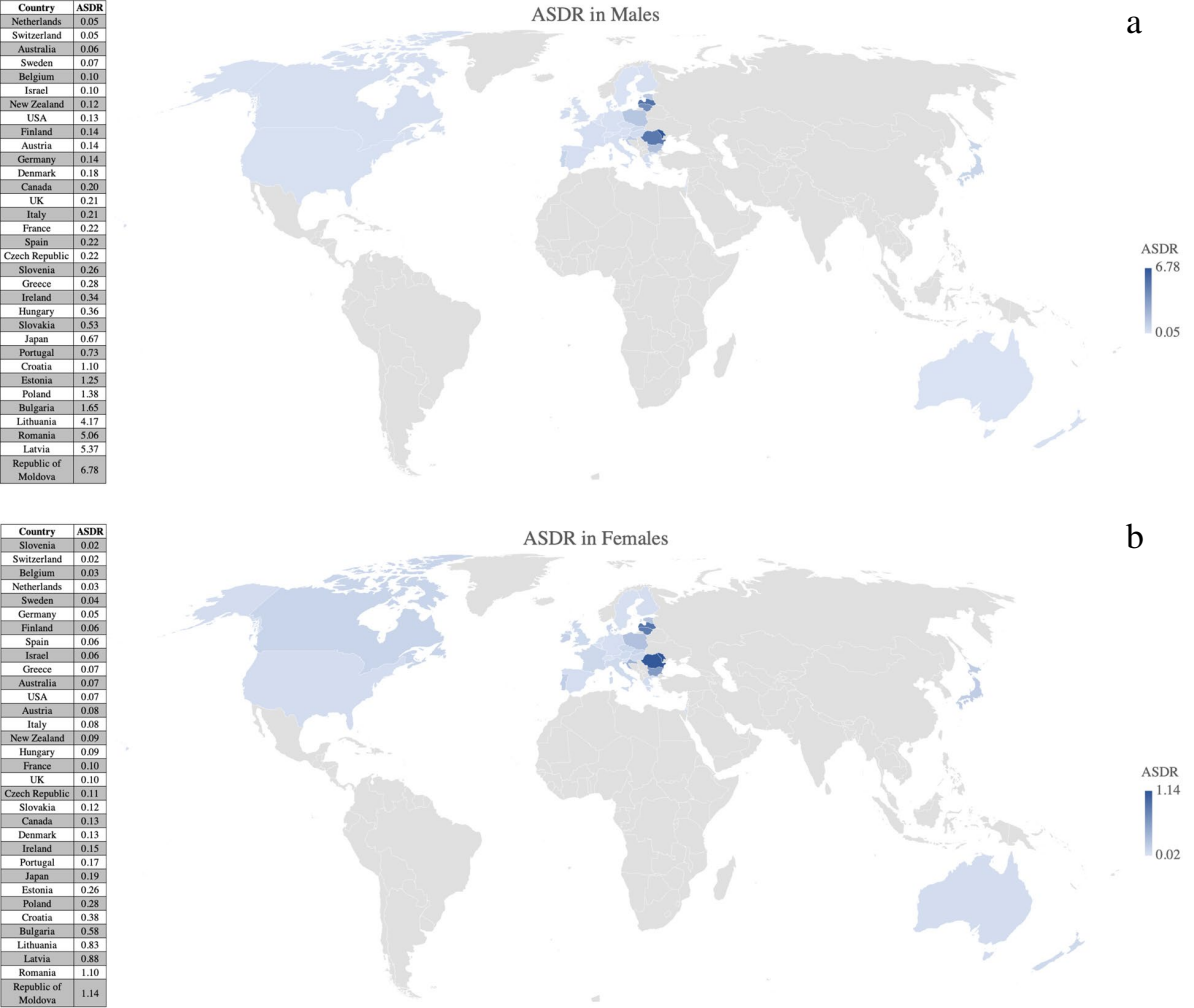
**A** & **B **Layered geographical heatmaps reporting tuberculosis related age standardized death rates per 100,000  in males and females for last study year

Table 1 and Figs. 2A, B and 3A, B show pulmonary TB mortality at the beginning and the end of the study period. Overall, we observed that all countries showed a decrease in mortality in both males and females except the Republic of Moldova, which showed an increase in females’ ASDR with absolute change (AC) of + 0.12 per 100,000 population. Among all 33 countries, Lithuania had the largest negative AC in male mortality (-12.01) between 1993–2018, and Hungary had the largest negative AC in female mortality (-1.57) between 1985–2017. The Netherlands had the smallest negative AC in male mortality (-0.13) between 1985–2017, and Denmark had the smallest negative AC in female mortality (-0.02) between 1994–2015.

Region-wise, in the Americas, only two countries met inclusion definition of data completeness. In the US, AC was observed to be -0.61 per 100,000 population in male mortality and (-0.17) in female mortality from 1985 through 2007. In Canada, AC was observed to be (-0.27) in male mortality and (-0.07) in female mortality between 1985–2005.

In the Europe region, the largest negative AC in male mortality was observed in Lithuania (-12.01) between 1993–2018, followed by Estonia (-10.67) between 1994–2016 and Latvia (-8.92) between 1996–2015. The largest negative AC in female mortality was observed in Hungary (-1.57) between 2004–2017, followed by Slovenia (-1.48) between 1985–2017 and Lithuania (-1.45) between 1993–2018. The only positive AC was observed in female mortality (+ 0.12) in the Republic of Moldova between 1991–2018.

In the Western Pacific region, Japan observed the largest negative AC in both male (-4.3 per 100,000 population) and female (-1.15) mortality between 1985–2017. Australia observed the smallest AC in both male (-0.17) and female (-0.11) mortality between 1985–2017. New Zealand observed a PC of (-0.56) in male mortality and (-0.26) in female mortality between 1985–2015.

### Joinpoint regression for changes in trends

**Fig. 4:**
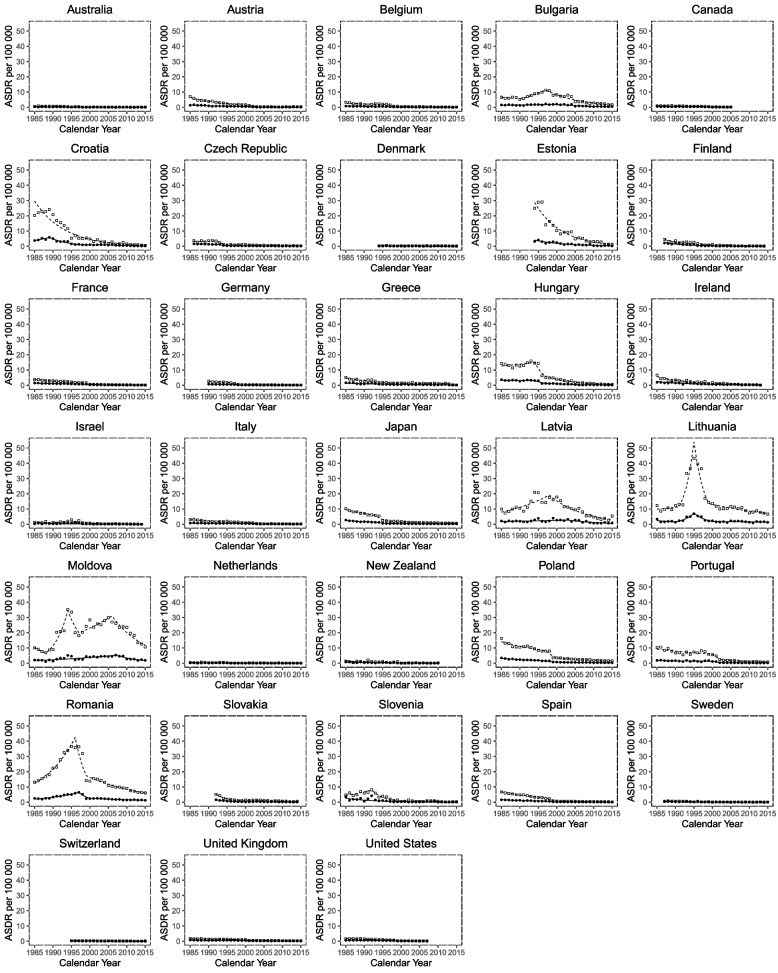
Joinpoint trends of  tuberculosis related age-standardized death rates per 100 000 population. Squares indicate male mortality, whereas circles indicate females

Table 2 and Fig. 4 show Joinpoint analysis of pulmonary TB mortality in males from 1985 to 2018. We report significant trend changes in the ASDR for the periods covered by each trend.

**Table 2:**
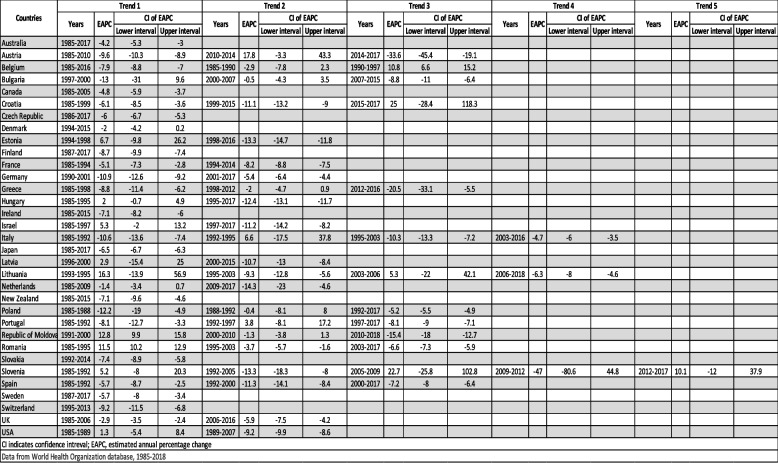
Joinpoint analysis of tuberculosis-related age standardized death rates per 100,000 population in males, for years 1985 to 2018, where data available. CI, confidence interval; EAPC, estimated annual percentage change

Slovenia had the most rapid recent mortality decline with an EAPC of -47% (2003–2016), followed by Australia (-33.6%, 2014–2017) and Greece (-20.5%, 2012–2016). Countries with trends showing increasing mortality include Croatia with an EAPC of + 25.0% between 2015–2017, followed by Slovenia (+ 22.7%, 2005–2009) and Austria (+ 17.8%, 2010–2014). Male mortality declined at a steady rate across the study period with only one Joinpoint in countries including Australia, Canada, Czech Republic, Denmark, Finland, Ireland, Japan, New Zealand, Slovakia, Sweden, and Switzerland showed declining mortality. Slovenia had the most Joinpoints, a total of five; with first period of increasing mortality between 1985–1992, then decreasing between 1992–2005, then increasing again between 2005–2009, followed by the most rapidly declining mortality of -47% between 2009–2012 and the most recent increasing mortality between 2012–2018. Both Lithuania and Italy had 4 Joinpoints. Italy started with declining mortality between 1985–1992, then increasing between 1992–1995, and then back to a steady decline between 1995–2003 and 2003–2016. Lithuania had increasing mortality between 1993–1995, then decreasing between 1995–2003, followed by increasing again between 2003–2006 and then decreasing between 2006–2018. Ten countries had 3 Joinpoints. Five among these, including Austria, Bulgaria, Greece, Poland, and Spain showed an overall decreasing mortality trend. Romania and the Republic of Moldova showed an initial increase in mortality followed by a decrease over the next two trends. Belgium and Croatia showed decreasing mortality in the initial years of the investigation, followed by a subsequent upward trend. Portugal had a unique pattern of decreasing mortality in the initial trend (1985–1992), followed by increasing mortality in the middle trend (1992–1997) and finally decreasing again in the last trend. Out of the nine countries with 2 Joinpoints, five showed an initial increase in the first trend followed by a decrease in the second. These include Estonia, Hungary, Israel, Latvia, and the USA. The other four, including France, Germany, Netherland, and the UK, showed 2 trends of decreasing mortality.

Table 3 and Fig. 4 show Joinpoint analysis of TB mortality in females from 1985 to 2018. New Zealand showed the most rapid recent mortality decline (-47.2%, 1985–2015), followed by Italy (-16.9%, 2009–2013). Countries with trends showing increasing mortality include Latvia with an EAPC of + 31.0% between 1996–1999, followed by Croatia (+ 24.9%, 2014–2017) and Hungary (+ 22.6%, 2007–2010). Female mortality declined at a steady rate across the study period except for the Republic of Moldova. With only one Joinpoint, the following countries showed declining mortality: Australia, Austria, Belgium, Canada, Czech Republic, Denmark, Finland, France, Greece, Ireland, Israel, Lithuania, Netherland, New Zealand, Poland, Slovakia, Slovenia, Spain, Sweden, Switzerland, and the UK. Italy had the most Joinpoints, a total of 6. It showed an alternating pattern that began with an increasing trend, followed by decreasing, and so on, with the latest increasing trend between 2013–2016 with an EPAC of + 7.2%. Hungary had 5 Joinpoints. The first 3 trends showed a decline at different rates with an EPAC of -35.1% for the third trend between 2004–2007, followed by the only positive trend between 2007–2010 (+ 22.6%) and then another trend showing a decline (-20.0%, 2010–2017). Croatia was the only country with 4 trends and showed a wide variation between each. It started with an increasing trend (19.9%, 1985–1987), followed by a decreasing trend (-5.8%, 1987–2008), then a more rapid decline between 2008–2014 (-20.1%), and the most recent increasing trend (+ 24.9%, 2014–2017). Three countries had 3 trends, each with a different pattern. Germany showed 3 negative trends with different rates, with the most recent being the most negative (-14.7%, 2013–2017). The Republic of Moldova had a positive EAPC in the 2 trends (+ 16.3%, 1991–1999; + 3.6%, 1999–2007) followed by a negative EAPC in the last one (-12.4%, 2007–2018). Lastly, Romania had an initial declining trend, followed by an increasing trend, then again, a declining trend. Portugal and Japan had 2 trends, both showing declining mortality. On the other hand, Bulgaria, Estonia, Latvia, and the USA showed 2 trends: the first showing positive EAPC and the second showing a negative EAPC.

**Table 3:**
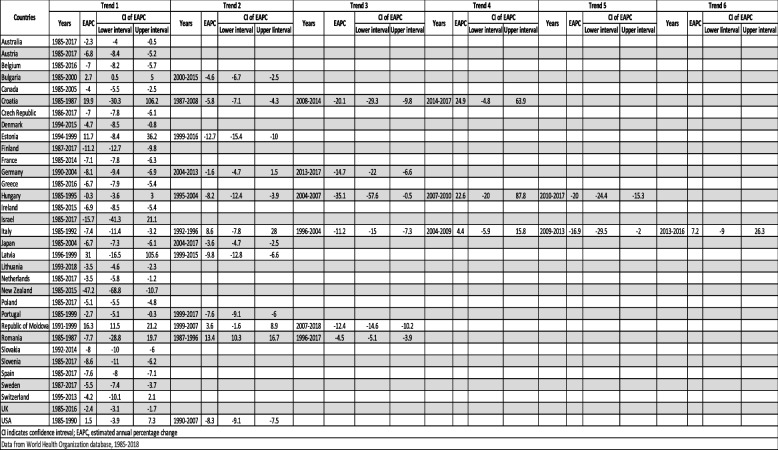
Joinpoint analysis for tuberculosis-related age standardized death rates per 100,000 population in  females, for years 1985 to 2018, where data available. CI, confidence interval; EAPC, estimated annual percentage change

## Post-hoc analysis

A strong negative correlation was observed between TB-related ASDRs and GDP, per capital health expenditure and SDI in both sexes. In males, the Pearson’s correlation coefficient with *p* values were: (per capita GDP, *r* = -0.570; *p* = 0.001), (per capita health expenditure, *r* = -0.581; *p* = 0.001), and (SDI, r = -0.522; *p* = 0.002). In females, the Pearson’s correlation coefficient with *p* values were: (per capita GDP, *r* = -0.592; *p* = 0.001), (per capita health expenditure, *r* = -0.595; *p* = 0.001), and (SDI, *r* = -0.541; *p* = 0.001). For countries with ASDRs > 1 per 100,000 in males and > 0.25 in females at the end of the study period the mean per capita GDP, per capita health expenditure, and SDI were $12,599 (SD, 5124), $571 (SD, 259), and 0.780 (SD, 0.47) respectively. For countries with ASDRs < 1 per 100,000 in males and < 0.25 in females the mean per capita GDP, per capita health expenditure, and SDI were $40,112 (SD, 16,290), $2709 (SD, 1193), and 0.832 (SD, 0.42) respectively. Both cohorts reached statistical significance for all three variables with *p*-values < 0.05 (supplementary table 1).

## Discussion

### Principal findings

In this observational study, we observed a decline in pulmonary TB related mortality individually in both sexes in all countries except among females in the Republic of Moldova. The biggest decline among males was observed in Lithuania, followed by Estonia, and Latvia. The biggest decline among females was observed in Hungary, followed by Slovenia, and Lithuania. The Republic of Moldova had the highest ASDR among males as well as females at the end of the observation period, followed among males by Latvia, Romania, and Lithuania and among females by Romania, Latvia, and Lithuania. Mortality among males was higher than females in all countries except Australia at the end of the observation period.

The 2020 Global TB Report published by the WHO estimated 10 million new TB cases worldwide in 2019. Of those, 8.2% were among people living with HIV. Most of the cases in 2019 occurred in the low-income countries in WHO regions of South-East Asia (44%), Africa (25%), and the Western Pacific (18%). Smaller proportions of cases occurred in the WHO regions of the Eastern Mediterranean (8.2%), the Americas (2.9%), and Europe (2.5%). Even in high-income countries with low-TB incidence, TB has reemerged as an important public health problem, mainly because of cases among immigrants from high-incidence countries and among marginalized populations [11].

### Socio-economic factors

The US, Canada, Australia, New Zealand, and most Western European countries had an ASDR < 0.5 in males and < 0.15 in females at the end of the observation period, while eight Central and Eastern European (CEE) countries, including the Republic of Moldova, Latvia, Romania, Lithuania, Bulgaria, Poland, Estonia, and Croatia had an ASDR > 1 in males and > 0.25 in females. Reasons for this significant disparity in pulmonary TB mortality are manifold, although socioeconomic factors appear to play a role. Interestingly, these eight countries also rank among the bottom third of the total 33 countries included in our study based on per capita gross domestic product at purchasing power parity (PPP) [12], as well as per capita health expenditure [13, 14]. Previous studies have shown that a lower socioeconomic status is associated with higher mortality among TB patients even in high-income countries [15–18]. Some of the factors that have traditionally been associated with TB include poverty, crowding, undernutrition, inadequate access to medical care, low literacy, unemployment, public assistance, social protection, and indoor air pollution. [19–21]. The United Nations included that no TB-affected household should experience catastrophic costs due to the disease by 2020 as one of the three Sustainable Development Goals (2016–2030)[22–24]. Since 2015, 17 countries have completed a national survey of costs faced by TB patients and their households and found that on average, 49% of people with TB and their families faced catastrophic costs (range: 19–83%). For people with drug-resistant TB, the figure was higher still, at 80% (range: 67–100%) [1].

The pivotal role of socioeconomic development and social protection in controlling the TB pandemic is emphasized by the rapid decline in the number of TB cases and deaths observed in Western Europe and North America around the turn of the twentieth century as incomes grew and housing and nutrition improved, and in the 1950s and 1960s, in the context of progress towards universal health coverage [25–27]. Early 2000 saw economic integration among European countries, with Latvia, Lithuania, Estonia, and Hungary joining the EU in 2004, Romania in 2007, and Croatia in 2013. Economic prosperity leading to improved socioeconomic conditions and enhanced living standards could have contributed to the continued decline in mortality observed in these countries. The Republic of Moldova, which is not a member of the EU, serves as a contrast. It holds the unique distinction of being the country with the highest mortality rates for both males and females at the end of the observation period and being the only country that observed an increase in mortality among females, and at the same time being the country with the lowest per capita gross domestic product (GDP) and lowest per capita health expenditure. It also has a high MDR-TB burden, with an estimated 1 in every 3 new TB cases being MDR-TB [1].

Additional challenges faced by CEE countries include a weak public health infrastructure. Furthermore, most CEE countries implement TB control services through specialized network facilities staffed by TB doctors and nurses with limited involvement of the primary health care services [28, 29]. Globally in 2019, an estimated 3.3% of new cases and 18% of previously treated cases had MDR/Rifampin-resistant TB (RR-TB), with the highest proportions in several countries of the former Soviet Union (above 20% in new cases and above 50% in previously treated cases) [1]. Similar results were seen in our study with high rates found in post-soviet countries like Estonia (males 1.25, females 0.26), Latvia (males 5.37, females 0.88), Lithuania (males 4.17, females 0.83), Republic of Moldova (males 6.78, females 1.14). WHO has now updated the Standardized shorter MDR-TB regimen based on results from several clinical trials [30–33]. The persistence of multidrug-resistant TB, and the emergence of extensively drug-resistant TB [34]. is a growing global public health concern [23].

### Strengths and limitations

The strengths of this investigation include the use of annual mortality data collected from national surveillance statistics from the WHO. These data have made it possible to assess population-level trends over an extended observation period, allowing comparisons in trends rather than absolute annual mortality rates. Despite this study’s strengths, there are a number of limitations that should be considered when interpreting the results. The data of US and Canada was available till 2007 and 2005 respectively. However, one notable strength of using longitudinal data is the ability to observe overall trends within individual countries after standardization and reporting these differences between health systems. Second, we did not attempt to assess the prevalence of morbidity associated with tuberculosis as our primary aim was to better understand changes in mortality trends; there may be substantial differences in the prevalence of TB between countries that we cannot elucidate in this current report. Third, TB mortality among HIV-positive persons is hard to measure, even when Vital registration (VR) systems are in place because deaths among HIV-positive persons are coded as HIV deaths, and contributory causes (e.g., TB) are often not reliably assessed or recorded. To achieve comparability over time, WHO has tried to standardize ICD-10 coding for HIV with detailed guidelines [35]. TB deaths among HIV-positive persons are estimated by WHO as either direct measurements from VR or indirect estimates from multiplying estimates of TB incidence by the estimates of the case fatality rate [36]. This would be an area of recommended future research. Finally, as with any observational study, causal statements regarding the observed trends cannot be made. The discussion is provided to assist future researchers, policymakers, and public health experts in focusing their efforts.

## Conclusions

Pulmonary TB mortality is disproportionately higher among Central and Eastern European countries. This communicable disease cannot be eliminated from any one region without a global approach. Priority action areas include ensuring early diagnosis and successful treatment to the most vulnerable groups such as people of foreign origin from countries with high burden of TB and incarcerated population. Incomplete reporting of TB-related epidemiological data to WHO excluded high-burden countries and limited our study to 33 countries only. Improvement in reporting is crucial to accurately identify changes in epidemiology, the effect of new treatments, and management approaches.

## Supplementary Information

**Additional file 1:**

## Data Availability

The datasets generated and/or analyzed during the current study are available in the World Health Organization repository, [https://www.who.int/data/gho]. The datasets used and/or analyzed during the current study available from the corresponding author on reasonable request.

## Abbreviations

AC: Absolute change
AIDS: Acquired immunodeficiency syndrome
ASDRs: Age standardized death rates
CEE: Central and Eastern European
DOTS: Directly observed treatment, short-course
EAPCs: Estimated annual percentage changes
GDP: Gross domestic product
HIV: Human immunodeficiency virus
LTBI: Latent TB infection
MDR TB: Multidrug-resistant tuberculosis
PC: Percentage change
PPP: Purchasing power parity
RR-TB: Rifampin-resistant TB
SD: Standard deviation
SDI: Sociodemographic index
TB: Tuberculosis
US: United States
VR: Vital registration
WHO: World Health Organization
