# Supplementary material for: Trends in pulmonary tuberculosis mortality between 1985 and 2018: an observational analysis
Source: BMC Pulm Med. 2023 May 26;23:184. doi: 10.1186/s12890-023-02458-9 (PMC10224609; doi:10.1186/s12890-023-02458-9)
Supplement: Supplementary file 1 — Additional file 1: [file 12890_2023_2458_MOESM1_ESM.docx]

Supplementary table 1: Per capita gross domestic product (US dollars), per capita health expenditure (US dollars), and sociodemographic index for the last study year by each country
